# Does an Adolescent’s Accuracy of Recall Improve with a Second 24-h Dietary Recall?

**DOI:** 10.3390/nu7053557

**Published:** 2015-05-13

**Authors:** Deborah A. Kerr, Janine L. Wright, Satvinder S. Dhaliwal, Carol J. Boushey

**Affiliations:** 1 School of Public Health, Curtin University, Perth, WA 6102, Australia; E-Mails: J.Wright@exchange.curtin.edu.au (J.L.W.); S.Dhaliwal@curtin.edu.au (S.S.D.); 2 Epidemiology Program, University of Hawaii Cancer Centre, Honolulu, HI 96844, USA; E-Mail: CJBoushey@cc.hawaii.edu; 3 Department of Nutrition Science, Purdue University, West Lafayette, IN 47907, USA

**Keywords:** diet, adolescent, 24-h dietary recall, dietary assessment, 5-step multiple-pass method

## Abstract

The multiple-pass 24-h dietary recall is used in most national dietary surveys. Our purpose was to assess if adolescents’ accuracy of recall improved when a 5-step multiple-pass 24-h recall was repeated. Participants (*n* = 24), were Chinese-American youths aged between 11 and 15 years and lived in a supervised environment as part of a metabolic feeding study. The 24-h recalls were conducted on two occasions during the first five days of the study. The four steps (quick list; forgotten foods; time and eating occasion; detailed description of the food/beverage) of the 24-h recall were assessed for matches by category. Differences were observed in the matching for the time and occasion step (*p* < 0.01), detailed description (*p* < 0.05) and portion size matching (*p* < 0.05). Omission rates were higher for the second recall (*p* < 0.05 quick list; *p* < 0.01 forgotten foods). The adolescents over-estimated energy intake on the first (11.3% ± 22.5%; *p* < 0.05) and second recall (10.1% ± 20.8%) compared with the known food and beverage items. These results suggest that the adolescents’ accuracy to recall food items declined with a second 24-h recall when repeated over two non-consecutive days.

## 1. Introduction

The evidence linking the adolescent diet with risk for chronic diseases later in life, including obesity and some cancers continues to increase [1,2]. This makes adolescents an important target group however, assessing diet in this age group is challenging. The methods most commonly used to evaluate diet in adolescents are dietary records, the 24-h dietary recall and food frequency questionnaires. However, acceptability of these methods by adolescents is not ideal [3]. Early adolescents, ages 11 to 14 years, in particular, are in that period of time when the novelty and curiosity of self-reporting food intakes starts to wane and the assistance from parents is seen as an intrusion [4].

The 24-h dietary recall is the method used in most national dietary surveys and has been recommended for use in European children aged 7 to 14 years [5]. The Food Surveys Research Group (FSRG) of the United States Department of Agriculture (USDA) has devoted considerable effort to improving the accuracy of the 24-h recall through development and refinement of the multiple-pass method. The 5-step multiple-pass method provides a structured interview format with specific probes and involves five structured sets of probing [6]. As the 24-h recall is conducted by interview, this may be less burdensome to participants, compared to other methods such as dietary records [7].

In children under 11 years comparisons of the 24-h recall with energy expenditure, as measured by doubly labelled water (DLW) show mixed results. One study showed a 14% greater energy intake than DLW estimated energy expenditure [8] and another showed only group estimates of energy intake as being valid [9]. An automated self-administered web version has been developed and is still undergoing evaluation in comparison to interviewer-administered 24-h recall [10].

The most common method of evaluating the accuracy of the multiple-pass 24-h recall with children is through observation of school meals comparing foods recalled with foods either observed as eaten or foods actually weighed [11,12]. These recalls have demonstrated both under-reporting and over-reporting, and incorrect identification of foods and rely on the dietitian being able to accurately assess the food type and quantity. A controlled feeding study offers a unique opportunity to assess the accuracy of dietary assessment. In a controlled feeding study, as the food and nutrient intake is known, it does not require the participants to be observed to confirm food and beverages consumed. There are occasions when adolescents are exposed to foods unfamiliar to them (e.g., new school lunch menus) and the controlled feeding study can duplicate this type of environment. The purpose of the study was to assess if adolescents’ accuracy of recall improved when a 5-step multiple-pass method for 24-h recall was repeated. Our hypothesis was that adolescents’ accuracy would improve when the 24-h recall was repeated. In addition, we determined the rates of intrusions and omissions and if these differed between the repeated recalls.

## 2. Methods

### 2.1. Participants and Study Design

Thirty-one Chinese-American boys and girls (11–15 years) were recruited to participate in a 7-week metabolic study where they lived in a campus residence hall facility converted into a metabolic unit for two 3-week balances in the summer separated by a 1-week washout when they returned to their homes [3,13]. During the balance, participants were scheduled for a variety of educational and recreational activities coordinated as a summer-camp environment. The participants were required to eat all meals, snacks and beverages provided. A camp supervisor sat at the table with a group of four to five participants for all meals and snacks and ensured that all food and beverages were consumed. On days two and five of the first week, 24 participants participated in the 24-h recall for both days and six participants participated in the 24-h recall for at least one of the two days. Only the 24 participants (11 boys and 13 girls) who completed two 24-h recall were included in the final analysis. Some participants were unavailable to complete the second recall due to other research commitments. The menu was a 4-day cycle and items were selected to reflect foods commonly eaten by Chinese-American youth. Participants did not know in advance what was to be served (Table 1). All food and beverages served were weighed and participants were required to consume all foods and beverages served. The study was approved by the Purdue University Institutional Review Board.

**Table 1:** Number and percentage of participants (*n* = 24) who reported omissions for each menu item in the first and second 24-h dietary recalls.

Eating occasion	1st recalled day menu	*n* (%) omissions ^a^	2nd recalled day menu	*n* (%) omissions
Breakfast	Pillsbury biscuit	0	Frosted flakes cereal	1 (4%)
	Jam (in packet)	2 (8%)	Milk	0
	Margarine (in packet)	2 (8%)	Sliced pears	5 (21%)
	Fruit cup	10 (42%)		
	Milk	4 (17%)		
Lunch	Turkey sandwich	1 (4%)	Hot dog (with bun)	2 (8%)
	Shrimp chips	2 (8%)	Steak fries	7 (29%)
	Sliced apples	2 (8%)	Ketchup (in packet)	2 (8%)
	Orange juice	0	Grapes	9 (38%)
			Orange juice	2 (8%)
Snack	Oatmeal cookies	4 (17%)	Gummi savers confectionary	7 (29%)
Dinner	Chicken thigh	0	Pasta	1 (4%)
	Orange marmalade sauce	2 (8%)	Pork & vegetable stir fry	5 (21%)
	Sliced carrots	3 (13%)	Pineapple pieces	10 (42%)
	Margarine (in packet)	13 (54%)	Milk	18 (75%)
	Rice	1 (4%)	Orange juice	1 (4%)
	Orange juice	3 (13%)		
Snack	Juice bar	0	Flavor pop	1 (4%)
	Orange juice	10 (42%)	Orange juice	5 (21%)

^a^: refers to the number of participants reporting omission for each food item.

### 2.2. 24-h Dietary Recall

Dietitians completed training in the 5-pass 24-h recall method prior to commencement of the study. Seven dietitians conducted the 24-h recalls, with each individual dietitian conducting between two and three 24-h recalls on each occasion. The allocation of dietitian to participant was based on availability. The dietitians were not familiar with the menu and were not present at any meals or snacks prior to the data collection. By ensuring that the dietitians did not view any of the meals consumed reduced the likelihood of bias or prompting due to knowing the menu when interviewing. A standard interview protocol was followed with each dietitian having a standard set of aids for portion size estimation. The target period for the interview was a recall of the previous day’s intake. Each recall covered a different menu day. To standardize the time of recall, the interviews took place following lunch and before dinner (commencing at 12:30 and finishing at 6:00 pm). The time taken for each participant to complete the 24-h recall on two separate occasions was recorded.

The procedure for the 5-step multiple-pass method was as previously described [14]. In the first step, participants were asked to provide a ‘quick list’ where they listed all foods and beverages consumed without interruption. In the second step, they were asked about “forgotten foods” where the interviewer followed a standard set of probes. The third step detailed “time and eating occasion” for each food item identified. In the fourth step, known as the “detail cycle,” standard probes were followed to obtain detailed information on the food and drinks consumed as well as how much they ate or drank. The fifth step was a “final review probe” for any forgotten food or beverage items.

### 2.3. Analyses

Key errors in the 5-step multiple-pass method were assessed with a separate score (number of correct matches divided by the number of known food and beverages) for the first four steps (quick list; forgotten foods; time and eating occasion; detailed description of the food/beverage) of the 24-h recall. A higher percent value was a higher score which translated to a better match. For the quick list and forgotten foods steps, broad food group category matches were identified. For example, the following responses were considered a match: if “milk” was recalled and “low-fat milk” was served, if “a turkey sandwich” was recalled and a “turkey sandwich with wheat bread and mayonnaise” was served. A score of 2 was assigned for a match, 1 for a partial match and 0 for an omission. For the time and eating occasion step, the food or beverage recalled needed to be identified as being consumed at the correct meal or snack and correct time. Matching in the detail step was split into the description and portion size matching. To be scored as a complete match, the food items needed to be recalled in detail. For example, in the turkey sandwich, single items needed to be recalled such as sliced turkey, wheat bread and mayonnaise to achieve a maximum score of 2. Foods were classified as partial matches when the recalled item was in the same family of foods. For example, if chicken was recalled instead of turkey a score of 1 was assigned. When the foods recalled could not be matched within the same food grouping, they were considered a mismatch and scored 0. For example, milk was recalled and orange juice was served. The food and beverage items that matched on portion size within ±10% of the weight or household measure were scored as a match (score = 3); within 10%–25% a partial match (score = 2), 25% or more was scored as a mismatch (score = 1) and missing or an omission scored as 0. The score for each step were summed, divided by the total possible score and expressed as a percentage, where a score of 100% indicated a perfect match for that particular step.

Omission and intrusion rates were assessed separately for the quick list and forgotten foods steps for the first and second recall. Food or beverages recalled by the participant but not served were classified as “intrusions” and foods or beverages served that were not recalled were classified as “omissions”. As only a few food or beverage items were recalled in the final probe these were included in the ‘forgotten food steps’ calculations. Weighted intrusion and omission rates were calculated according to the method outlined by Baxter *et al.* [15] and were calculated as follows: Omission rate = (sum of weighted omissions/[sum of weighted omissions + sum of weighted matches]) × 100%. Values range from 0% (no omissions) to 100% (no food or beverage items reported eaten). A weight was assigned to each item according to importance by meal component. For example turkey sandwich = 2; condiment such as jam, margarine = 0.33; other meal/snack single items = 1. Intrusion rate (percentage of items reported eaten but not served) was calculated as (sum of weight intrusions/[sum of weighted intrusions + sum of weighted matches]) × 100%. Values range from 0% (no intrusions) to 100%. Means and standard deviations for omission and intrusion rates expressed as percentages were determined for the first and second dietary recall for the quick list and the forgotten foods steps. The score for each defined component for four of the five steps were expressed as a percentage, as well as the omission rate and the intrusion rate. Non-parametric statistics were used to test the difference in scores and rates between the recalled days. Wilcoxon Signed Rank Test (with exact test option) were used to assess if there were significant differences in the components and rates comparing the first and second recall.

The participants’ 24-h recalls and the known food and beverage items for the days recalled, were analyzed using the Nutrition Coordinating Center food and nutrient database. Data coding and entry were performed by staff trained in the use of the Nutrition Data System for Research (NDS-R) Database Version v5.0/35 (^©^ Regents of the University of Minnesota). Accuracy of the 24-h recalls was assessed by comparing the items recalled by the adolescents with the known food and beverage items. A paired sample t-test was used to test differences between the known food and beverage items and foods recalled.

## 3. Results

There were 24 participants who completed two 24-h recalls. The 11 boys mean (±standard deviation) ages were 13.9 ± 1.2 years with a mean body mass index (BMI) of 20.0 ± 4.1. For the 13 girls, these same parameters were 13.2 ± 1.3 years old and 19.2 ± 2 for BMI.

The number of participants who reported omissions for each menu item is shown in Table 1. For the first recall, the most frequently omitted item was margarine, where 13 participants (out of 24) did not recall the item. In the second recall, milk was omitted by 18 participants (75%). 

Table 2 shows the individual details of the intrusions (food or beverages recalled but not served). Ten participants reported intrusions on the first recall compared with 16 intrusions on the second recall. There were five children out of 24, who reported no intrusions for either the first or the second recall.

**Table 2:** Details of food and beverage intrusions reported by participants (*n* = 24) in either the first, second, or both 24-h dietary recalls. Intrusions were food or beverage items recalled but not served.

	Intrusions	
**Participant**	**1st recall**	**2nd recall**
1	0	0
2	0	orange juice
3	0	butter, milk, orange juice
4	0	orange juice
5	orange juice	0
6	0	0
7	apples	0
8	0	0
9	shrimp chips, orange juice	orange juice, pears
10	orange juice, apples	orange juice, orange juice, catsup
11	0	0
12	0	milk
13	0	0
14	peanut butter sandwich, jam	peaches
15	milk	butter, jelly, biscuit
16	milk	milk
17	0	milk
18	0	orange juice
19	orange juice	juice box
20	orange juice, popsicle	peanut butter, crackers, popsicle
21	soup	0
22	0	orange juice
23	0	orange juice
24	0	Juice box
Participants reporting intrusions	10	16

Table 3 shows the component scores for the first four steps of the 24-h recall expressed as a percentage where a higher score indicates a better food and beverage match. Examination of the scores for each component comparing the first recall with the second recall showed significant differences in the matching for the time and occasion step (*p* < 0.01), detailed description (*p* < 0.05) and portion size matching (*p* < 0.05), but not for the first (quick list) or second (forgotten foods) step.

**Table 3:** Data from 24-h dietary recalls collected from adolescents (*n* = 24): The component scores of the first four steps of the 5-step multiple-pass method by first and second day of recall. For Step 4, the description and portion size are presented separately. A higher score indicates a better match between foods and beverages recalled and the known foods and beverages consumed.

	Step 1: Quick List	Step 2: Forgotten Foods	Step 3 : Time and Eating Occasion	Step 4: Detail Cycle	
				Description	Portion Size
	$\overset{\;}{\leftarrow }$	Mean ± Standard Deviation (%)		$\overset{\;}{\to }$	
First recall	75 ± 15	84 ± 10	84 ± 11	79 ± 10	61 ± 10
Second recall	71 ± 20	80 ± 12	76 ± 13 **	72 ± 11 *	55 ± 9 *

* *p* < 0.05; ** *p* < 0.01 comparing the first recall with the second recall.

The rate of omissions and intrusions were compared between the first and second recall for the first two steps of the 24-h recall (Table 4). For both the first and second recalls, the omission rate decreased significantly from the ‘quick list’ to the ‘forgotten foods’ step, *i.e.*, first recall was 21.9 ± 13.9% to 12.9 ± 8.3% (*p* < 0.001) and the second recall was 28.9 ± 21.3% to 20.5 ± 14.1% (*p* < 0.001). This indicates the importance of the second step in the 24-h recall for improving recalled items. Lower omission rates were observed for the first recall compared with the second recall, for both the ‘quick list’ (*p* < 0.05) and the ‘forgotten foods’ step (*p* < 0.01). In the second recall, intrusion rate increased from the first to the second step (2.8 ± 4.4% to 6.4 ± 5.5% respectively; *p* < 0.001), indicating more errors occurred with food and beverages recalled but not served. The time taken to complete the first recall was longer compared to the second recall (51 ± 13 min to 35 ± 11 min respectively; *p* < 0.01).

**Table 4:** Omission and intrusion rates by the first and second day of recall for the Quick List and Forgotten Foods Steps of the 5-step multiple-pass method completed by 24 adolescents.

Recall	Step	Omission Rate (%) ^a^ *n* = 24	Intrusion Rate (%) ^b^ *n* = 24
First Recall		Mean ± Standard Deviation	
	Quick List	21.9 ± 13.9	2.8 ± 5.4
	Forgotten Foods	12.9 ± 8.3 ***	4.3 ± 6.2
Second Recall	Quick List	28.9 ± 21.3 *	2.8 ± 4.4
	Forgotten Foods	20.5 ± 14.1 ***, **	6.4 ± 5.5 ***

^a^: Omission rate = (sum of weighted omissions/[sum of weighted omissions + sum of weighted matches]) × 100%. Values range from 0% (no omissions) to 100% (no food or beverage items reported eaten); ^b^: Intrusion rate (percentage of items reported eaten but not served) was calculated as (sum of weight intrusions/[sum of weighted intrusions + sum of weighted matches]) × 100%. Values range from 0% (no intrusions) to 100%; ***: *p* < 0.001 comparing Quick List with Forgotten Foods; *: *p* < 0.05 comparing first recall with the second recall for Quick List; **: *p* < 0.01 comparing first recall with the second recall for Forgotten Foods.

Table 5 shows the differences between the nutrient composition of the known food and beverage items consumed and the 24-h dietary recall for the first and second day of recall. For energy intake the adolescents were able to accurately recall food and beverage items. For the first recall there was an over-estimate of energy (11.3 ± 22.5%; *p* < 0.05), protein (17.2 ± 29.4%; *p* < 0.05) and fat intake (35.9 ± 45.9%; *p* < 0.01) compared with the known food and beverage items. There were also significant differences in calcium (*p* < 0.001), fiber (*p* < 0.01), and iron (*p* < 0.01), between the known food and beverage items and the first recall. In the second recall, there were differences between the known food and beverage items and the interviewer-conducted recall for calcium (22.1 ± 48.9%; *p* < 0.05) and folate (39.2 ± 32.5%; *p* < 0.001) only. The standard deviation for first recall and second recall, respectively, for energy was 375 kcal and 413 kcal. The standard deviation for the mean of the energy from first and second recall was 317 kcal consistent with the use of two recalls reducing the variability of the data.

**Table 5:** Mean differences between the nutrient composition of the food and beverages consumed (actual intake) and the 24-h dietary recall by the first and Second day of recall among adolescents participating in a controlled feeding study (*n* = 24). A negative value for % difference indicates an underestimation by the recall; a positive value indicates an overestimation by the recall.

	1st Recalled Day			2nd Recalled Day		
	**Actual intake**	**Recalled intake**	**% Difference**	**Actual intake**	**Recalled intake**	**% Difference**
Energy (kcal/day)	1699 ± 236	1877± 375	11.3 ± 22.5 *	1744 ± 194	1870 ± 413	10.1 ± 20.8
Protein (g/day)	58 ± 10	67 ± 18	17.2 ± 29.4 *	56 ± 7	54 ± 18	−2.9 ± 30.4
Carbohydrate (g/day)	294 ±36	300 ± 62	2.9 ± 22.7	305 ± 28	333 ± 79	9.2 ± 23.9
Fat (g/day)	36 ± 6	48 ± 17	35.9 ± 45.9 **	39 ± 7	40 ± 15	4.2 ± 38.7
Calcium (mg)	538 ± 32	889 ± 401	65.6 ± 71.7 ***	655 ± 5	801 ± 326	22.1 ± 48.9 *
Vitamin C (mg)	399 ± 16	381 ±156	−4.5 ± 39.1	426 ± 10	428 ± 141	0.5 ± 33.5
Total dietary fibre	16.8 ± 1.4	13.7 ± 3.8	−17.7 ± 24.7 **	13.3 ± 1.4	12.4 ± 4.8	−7.1 ± 35.6
Total folate (mcg)	660 ± 38	534 ± 193	−19.2 ± 28.4 **	732 ± 37	747 ± 160	1.9 ± 21
Iron (mg)	10.4 ± 1.7	10.2 ± 3.0	−1.6 ± 27.5	11.3 ± 1.0	15.8 ± 3.9	39.2 ± 32.5 ***
Zinc (mg)	6.4 ± 1.5	6.2 ± 1.7	−1.2 ± 26.1	5.9 ± 0.7	7.1 ± 3.2	19.6 ± 49
Macronutrient energy distribution						
Protein (%)	13.6 ± 0.7	14.4 ± 3.1	5.7 ± 20.8	12.7 ± 0.6	11.2 ± 2.8	−11.9 ± 22.7 *
Carbohydrate (%)	69.3 ± 1.9	63.0 ± 5.4	−9.2 ± 7.6 ***	70.1 ± 1.1	69.9 ± 7.9	−0.3 ± 11
Fat (%)	19.0 ± 0.8	22.6 ± 5.0	19.6 ± 26.8 **	19.9 ± 1.4	18.9 ± 5.9	−4.4 ± 30.7

*: *p* < 0.05 between recall and actual intake; **: *p* < 0.01; ***: *p* < 0.001.

## 4. Discussion

This study showed that that adolescents’ accuracy of recall did not improve when a 24-h recall was repeated. Accuracy was assessed by direct comparison of the known food and beverage items consumed with those recalled by the adolescents. However, the combined standard deviation for the mean of the energy of the two days indicates the overall variability in the estimates of energy intake would be reduced by the inclusion of a second recall. A unique aspect of this study was all food and beverage items were weighed prior to being offered. Thus the food and beverages recalled were known by the investigators, thus eliminating potential biases associated with observers. Overall, the omission rate was greater in the second recall compared to the first recall, which was offset by a significantly higher rate of intrusions. This partially explains the energy estimates from the two recalls being nearly identical (1877 ± 375 kcal and 1870 ± 413 kcal). The comparisons between the reported and known food and beverage items were statistically significantly different for only the first recall at 11% higher (*p* < 0.05) and 10% higher (NS) for the second recall. Thus, despite the errors (either omissions or intrusions) made by the adolescents, these had only a minor effect on the accuracy of estimated energy intake. However, although the overall energy intake may appear acceptable, the adolescents made reporting errors in foods omitted (Table 1), additional items recalled but not served (intrusions, Table 2) as well as errors in portion size estimation (Table 3) that affected the accuracy of the energy and nutrient intake. By using a controlled feeding study where the amount of food and beverage items served was known we were able to identify these errors. Of importance, these errors would not be detected with biomarkers such as doubly labelled water or urine nitrogen or by direct observation where the exact weight of the food or beverage was not known.

The omission rate, where foods were not recalled by the participant but were consumed, was shown to improve from the quick list (first step) to the forgotten foods (second step) on both recalls. This consistent improvement demonstrates the importance of these sequential steps in the multi-pass method. The omission rate for the second step increased from 13% in the first recall to 20% in the second recall. Intrusions (food or beverages recalled by the participant but not consumed) showed a significant increase from the first to the second step on the second recall. However, overall the rate of intrusions and omissions were lower than reported by Baxter *et al.* [15] who showed in 10 and 11 year olds an intrusion rate of 24% and an omission rate of 34% for recalls conducted on school lunches. Intrusions may be based on specific memories of foods consumed during the recording period or at a previous time [16]. As the 24-h recalls were conducted during a metabolic study, some of the foods or beverages may have been unfamiliar to the participants and the children would have been cognizant of the absence of their usual foods. The study design in the current study may be a similar situation to school lunches at the beginning of the school year or when menus items are changed. Thus, if there is a desire to assess children’s intakes at these times, these results would be most relevant. Further, the possibility exists that the recalls may have improved if the 24-h dietary recalls were conducted at the end of camp. On the other hand, frequency of foods being served didn’t necessarily help as demonstrated by the errors that occurred with the recall of milk and orange juice.

In meal observation studies, trained observers record the food and beverage items and estimate the portion sizes [11,17]. For a reference standard, these studies rely on the dietitian being able to unobtrusively assess the food type and quantity accurately. In the current study the dietitians conducting the 24-h recall did not observe the adolescents during meal and snack times, as the food and beverage items served were known and weighed, making accurate comparison to the recalled food and beverage items possible. Thus, some of the differences between this study and others regarding omission and intrusion rates may be due to study design and implementation.

There was poorer matching of portion size with the second recall (55% matching) compared to the first recall (61% matching; *p* < 0.05). Standard food models were used in the current study however, estimation accuracy of portion size decreased from the first recall to the second recall, indicating that repeating the task did not improve the estimation accuracy of portion sizes. Further, the more detailed description of foods and beverages declined between the first and the second recall (79% compared to 72%, *p* < 0.05). The participants’ scores, indicating better matches, did not improve for any of the steps with the second 24-h recall. The adolescents took significantly less time to complete the second recall which perhaps reflects less attention to detail instead of becoming more skilled with the process.

Ironically, despite the increase in the number of foods not matching the actual foods served for the second recall, the energy/nutrient profile better matched the energy/nutrient profile of the foods associated with the second recall than occurred with the first recall where there was a better food match. In a study among adults using an objective biomarker for energy [18], the second administration of the 24-h recall showed greater underreporting. Thus, the results among the adolescents in this study reflect the same observations made among adults of some sort of waning of enthusiasm to provide consistent quality data. On the other hand, the increase in intrusions, could reflect a desire among the adolescents to guess in order to provide a “right” answer [3]. In addition, the time taken to complete the second recall was shorter, perhaps indicating declining interest in the process. The observed decrease in time taken to complete the second recall compared to first was also observed among the adults in the OPEN study [18]. Although the adolescents in this study were able to complete the second recall more quickly they made more errors in recalling the food and beverage items and started to recall items they didn’t consume. This latter observation may reflect a social desirability bias [19]. Other possible factors proposed to influence reporting accuracy of the recalls have been noted by Baxter *et al.* [20] in (fourth-grade) 9 to 10 year old children, who found the retention interval between when the recall was conducted to be important. When the recall was conducted on the previous day’s intake errors (intrusions and omissions) were higher for the afternoon and evening. In the current study, the recall was conducted on the previous day’s intake and the interview undertaken at the same time for both recalls. Baxter *et al.* [20] also suggested that efforts to improve children’s accuracy should focus on the reporting food items as they found that when children accurately recall, the amounts reported are quite accurate. Milk and orange juice (Table 2) were common intrusions in the current study. As both of these items were served on several occasions on the menu, it may have led to more mistakes in recalling the items.

There are several limitations of the current study that may limit the generalizability of the findings. The relatively small sample size reduces the statistical power of the study. However, as the participants were part of a metabolic study, we were limited by the constraints of the primary study in the sample size. Future studies should seek to replicate these findings in a larger sample. Further the adolescents in the current study were of one ethnic group and part of a metabolic study where they were required to eat everything, which may not reflect what would occur in a community-dwelling situation. Finally, there may have been an order effect observed with the second recall, due to factors related to the camp environment. Focus group data suggests that adolescents find dietary recalls ‘pointless’ and ‘boring’ [3]. It may well be that dietary recalls were of less interest to the adolescents, as they took part in a variety of educational and recreational activities coordinated as a summer-camp environment and these may have had greater appeal.

## 5. Conclusions

These results of this study show that the adolescents’ accuracy to recall food items declined with a second recall. Our hypothesis was that adolescents’ accuracy would improve when the 24-h recall was repeated. This was not the case and errors occurred due to omissions, intrusions and portion size estimation. These results indicate that adolescents’ accuracy to recall food items declined when the 24-h recall was repeated over two non-consecutive days. Due to the limitations of the study, no recommendation can be made on whether a second recall will improve accuracy, but our findings suggest that further research in this age group is needed to insure accurate results. Dietary assessment methods need to continue to evolve to address these challenges as further improvements will enhance the consistency and strength of the association of diet with disease risk, especially in light of the current obesity epidemic among youth.

## References

[1] Ogden C.L., Carroll M.D., Kit B.K., Flegal K.M. (2012). Prevalence of obesity and trends in body mass index among us children and adolescents, 1999–2010. JAMA.

[2] Nimptsch K., Malik V.S., Fung T.T., Pischon T., Hu F.B., Willett W.C., Fuchs C.S., Ogino S., Chan A.T., Giovannucci E. (2014). Dietary patterns during high school and risk of colorectal adenoma in a cohort of middle-aged women. Int. J. Cancer.

[3] Boushey C.J., Kerr D.A., Wright J., Lutes K.D., Ebert D.S., Delp E.J. (2009). Use of technology in children’s dietary assessment. Eur. J. Clin. Nutr..

[4] Livingstone M.B., Black A.E. (2003). Markers of the validity of reported energy intake. J. Nutr..

[5] Andersen L.F., Lioret S., Brants H., Kaic-Rak A., de Boer E.J., Amiano P., Trolle E. (2011). Recommendations for a trans-european dietary assessment method in children between 4 and 14 years. Eur. J. Clin. Nutr..

[6] Conway J.M., Ingwersen L.A., Moshfegh A.J. (2004). Accuracy of dietary recall using the USDA five-step multiple-pass method in men: An observational validation study. J. Am. Diet. Assoc..

[7] Burrows T.L., Martin R.J., Collins C.E. (2010). A systematic review of the validity of dietary assessment methods in children when compared with the method of doubly labeled water. J. Am. Diet. Assoc..

[8] Fisher J.O., Johnson R.K., Lindquist C., Birch L.L., Goran M.I. (2000). Influence of body composition on the accuracy of reported energy intake in children. Obes. Res..

[9] Johnson R.K., Driscoll P., Goran M.I. (1996). Comparison of multiple-pass 24-hour recall estimates of energy intake with total energy expenditure determined by the doubly labeled water method in young children. J. Am. Diet. Assoc..

[10] Baranowski T., Islam N., Baranowski J., Martin S., Beltran A., Dadabhoy H., Adame S.H., Watson K.B., Thompson D., Cullen K.W. (2012). Comparison of a web-based *versus* traditional diet recall among children. J. Acad. Nutr. Diet..

[11] Baranowski T., Islam N., Baranowski J., Cullen K.W., Myres D., Marsh T., de Moor C. (2002). The food intake recording software system is valid among fourth-grade children. J. Am. Diet. Assoc..

[12] Baxter S.D., Hardin J.W., Smith A.F., Royer J.A., Guinn C.H., Mackelprang A.J. (2009). Twenty-four hour dietary recalls by fourth-grade children were not influenced by observations of school meals. J. Clin. Epidemiol..

[13] Wu L., Martin B.R., Braun M.M., Wastney M.E., McCabe G.P., McCabe L.D., DiMeglio L.A., Peacock M., Weaver C.M. (2010). Calcium requirements and metabolism in Chinese-American boys and girls. J. Bone Miner. Res..

[14] Conway J.M., Ingwersen L.A., Vinyard B.T., Moshfegh A.J. (2003). Effectiveness of the us department of agriculture 5-step multiple-pass method in assessing food intake in obese and nonobese women. Am. J. Clin. Nutr..

[15] Baxter S.D., Hitchcock D.B., Guinn C.H., Royer J.A., Wilson D.K., Pate R.R., McIver K.L., Dowda M. (2013). A pilot study of the effects of interview content, retention interval, and grade on accuracy of dietary information from children. J. Nutr. Educ. Behav..

[16] Smith A.F., Baxter S.D., Hardin J.W., Royer J.A., Guinn C.H. (2008). Some intrusions in dietary reports by fourth-grade children are based on specific memories: Data from a validation study of the effect of interview modality. Nutr. Res..

[17] Baxter S.D., Thompson W.O., Litaker M.S., Guinn C.H., Frye F.H.A., Baglio M.L., Shaffer N.M. (2003). Accuracy of fourth-graders’ dietary recalls of school breakfast and school lunch validated with observations: In-person *versus* telephone interviews. J. Nutr. Educ. Behav..

[18] Subar A.F., Kipnis V., Troiano R.P., Midthune D., Schoeller D.A., Bingham S., Sharbaugh C.O., Trabulsi J., Runswick S., Ballard-Barbash R. (2003). Using intake biomarkers to evaluate the extent of dietary misreporting in a large sample of adults: The open study. Am. J. Epidemiol..

[19] Guinn C.H., Baxter S.D., Royer J.A., Hardin J.W., Mackelprang A.J., Smith A.F. (2010). Fourth-grade children’s dietary recall accuracy for energy intake at school meals differs by social desirability and body mass index percentile in a study concerning retention interval. J. Health Psychol..

[20] Baxter S.D., Hardin J.W., Guinn C.H., Royer J.A., Mackelprang A.J., Smith A.F. (2009). Fourth-grade children’s dietary recall accuracy is influenced by retention interval (target period and interview time). J. Am. Diet. Assoc..

